# Two stages of parafoveal processing during reading: Evidence from a display change detection task

**DOI:** 10.3758/s13423-015-0995-0

**Published:** 2016-01-14

**Authors:** Bernhard Angele, Timothy J. Slattery, Keith Rayner

**Affiliations:** 1 Department of Psychology, Faculty of Science and Technology, Bournemouth University, Fern Barrow, Poole, BH125BB Dorset UK; 2 Department of Psychology, University of California San Diego, San Diego, CA USA

**Keywords:** Eye movements, Reading, Display changes, Gaze-contingent boundary paradigm, Display change detection

## Abstract

We used a display change detection paradigm (Slattery, Angele, & Rayner *Human Perception and Performance*, *37*, 1924–1938 2011) to investigate whether display change detection uses orthographic regularity and whether detection is affected by the processing difficulty of the word preceding the boundary that triggers the display change. Subjects were significantly more sensitive to display changes when the change was from a nonwordlike preview than when the change was from a wordlike preview, but the preview benefit effect on the target word was not affected by whether the preview was wordlike or nonwordlike. Additionally, we did not find any influence of preboundary word frequency on display change detection performance. Our results suggest that display change detection and lexical processing do not use the same cognitive mechanisms. We propose that parafoveal processing takes place in two stages: an early, orthography-based, preattentional stage, and a late, attention-dependent lexical access stage.

In reading research, preview benefit effects are highly reliable (for reviews, see Rayner, 1998, 2009; Schotter, Angele, & Rayner, 2012). When readers have a valid preview of the upcoming word (word *n* + 1), they subsequently look at it for 20–50 ms less than when they had an invalid preview. Virtually all of the research documenting the preview benefit has utilized the gaze-contingent boundary paradigm (Rayner, 1975), in which a preview stimulus changes to a target stimulus when the readers’ eyes cross an invisible boundary location. Because of saccadic suppression (Matin, 1974), readers are generally not aware of the change.

However, in every experiment, a small minority of readers are aware of display changes. White, Rayner, and Liversedge (2005) compared subjects (*n* = 16) who reported noticing display changes with those who did not (*n* = 32), and found that subjects who were aware of the changes produced a different pattern from those who were not. More recently, Slattery, Angele, and Rayner (2011) reported a more precise way of examining display change sensitivity using the signal detection paradigm (Macmillan & Creelman, 2005). After each trial, subjects indicated whether something had changed in the sentence they were reading (displayed in alternating case). Slattery et al. also varied when the change was triggered—immediately upon crossing the boundary, or delayed by 15–25 ms—replicating the findings (McConkie & Zola, 1979; Rayner, McConkie, & Zola, 1980) that readers were not aware of alternating-case changes (*gReEn→GrEeN*) across saccades and did not change their eye movement behavior when there was no display change delay. However, in the delayed condition, readers did notice the change, and their eye movement data (as per White et al., 2005) did differ from the immediate condition. Subjects’ sensitivity to display changes was related to fixation distance from the invalid preview prior to the display change, as well as to the precise timing of the display change relative to the start of the postchange fixation.

In the present experiment, we utilized Slattery et al.’s (2011) change detection paradigm to examine whether display change detection and lexical processing use the same cognitive resources. If so, we should find that display change detection effects mirror previous findings on the preview benefit (enhanced sensitivity following higher-frequency words). If different resources are used, display change detection and the preview benefit should be unrelated. Slattery et al. found some evidence that display change detection and word identification are related by examining different types of previews. When only letter case changed between the preview and target (e.g., *gReEn* to *GrEeN*), detection performance was poor as compared to when letter identities changed (e.g., *jNxVa* to *gReEn*). This agrees with previous evidence suggesting that readers quickly switch from a visual-form representation (in which *a*, *A*, a, and *A* are different letters) to a form-invariant, abstract letter code (McConkie & Zola, 1979; Rayner et al., 1980). Thus, display change detection tasks might prove useful in investigating when and how readers transition from visual-form representations to abstract letter codes.

However, there is an alternative explanation: Perhaps readers are sensitive to unusual letter sequences. There is ample evidence that ongoing foveal processing is influenced by the presence of nonwordlike letter strings in the parafovea (for a review, see Schotter et al., 2012). Angele, Tran, and Rayner (2013) found that foveal processing can be both inhibited by nonwordlike parafoveal letter strings and facilitated by parafoveal letter strings that are similar or identical to the foveal word. Readers may use the presence of nonwordlike parafoveal strings as an indicator of the presence of a display change, even without conscious awareness of the actual change. In order to test this hypothesis, we manipulated the parafoveal preview in the present study to be either identical to the word (e.g., *garden* for *garden*), wordlike (e.g., *puvtur* for *garden*), or nonwordlike (e.g., *xbtchp* for *garden*). Unlike Slattery et al. (2011), we did not use alternating cases, making our manipulation more naturalistic. If readers use letter identity to detect display changes, we should find no difference between the nonidentical preview conditions, since neither shares letter identities with the target. If, however, readers detect display changes by determining how wordlike the parafoveal preview is, we should see more accurate detection in the nonwordlike than in the wordlike preview condition.

Additionally, if display change detection uses the same resources as normal reading, it should be influenced by foveal processing difficulty. Processing a difficult word *n* in the fovea reduces parafoveal preprocessing of the upcoming word *n* + 1 (Henderson & Ferreira, 1990), and, correspondingly, the amount of preview benefit observed when fixating that word. Accordingly, we examined the sensitivity to detecting a display change when the preboundary words *n* were either high or low in frequency. If display change detection is driven by the same word identification processes responsible for the preview benefit, display change detection should be poorer when the preboundary word is low rather than high in frequency.

## Method

### Subjects

A group of 32 undergraduates at the University of California San Diego participated for course credit. All were native speakers of English, had normal or corrected-to-normal vision, and were naïve concerning the purpose of the experiment.

### Apparatus

An SR Research EyeLink 1000 eyetracker recorded subjects’ eye movements with a sampling rate of 2000 Hz. Sentences were displayed on an Iiyama VisionMaster Pro 454 video monitor with a refresh rate of 150 Hz. The viewing distance was approximately 60 cm, with 3.8 letters equaling one degree of visual angle.

### Materials and procedure

**Fig. 1:**
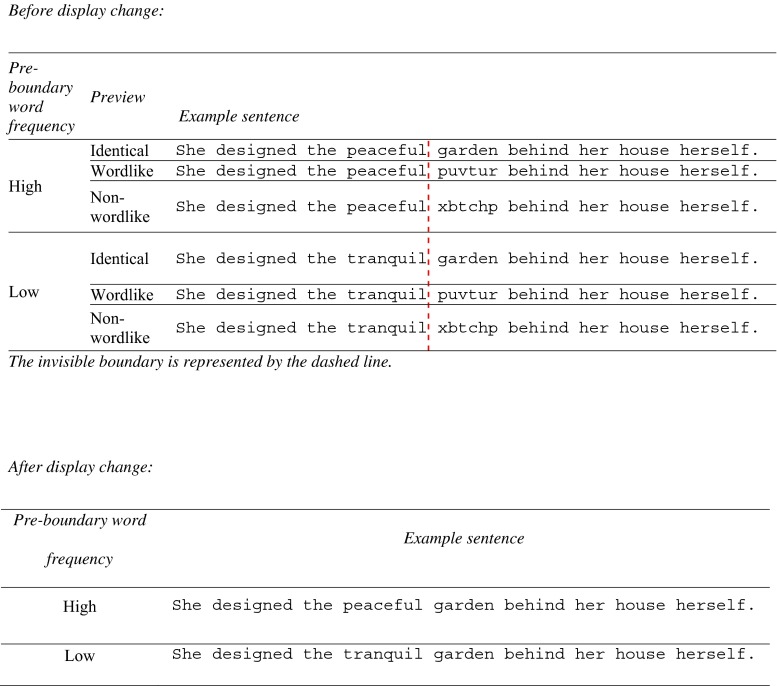
Example items

Participants read 102 experimental sentences and 30 filler sentences binocularly, but only their right eye movements were recorded. In each experimental sentence, an adjective (word *n*) was followed by a noun (word *n* + 1). These sentences were constructed so that word *n* could be of either high (*peaceful*) or low (*tranquil*) frequency (see Fig. 1). Word frequency estimates were computed using an unlemmatized list generated from the British National Corpus (Kilgarriff, 2006). Table 1 shows frequency, length, and mean log token bigram frequency estimates (the latter obtained from the *N*-Watch software; Davis, 2005).

**Table 1:** Mean preboundary and target word properties (*SD* in parentheses)

Stimulus	Frequency	Mean log bigram frequency	Length
Preboundary word (high-frequency condition)	210 (210)	2.9 (0.31)	5.4 (0.94)
Preboundary word (low-frequency condition)	4.2 (5.2)	2.5 (0.32)	5.4 (0.94)
Target word	150 (160)	2.9 (0.38)	5 (1.3)
Wordlike preview	nonword	1.2 (1.1)	5 (1.3)
Nonwordlike preview	nonword	–1.4 (0.95)	5 (1.3)

An invisible boundary was located between the last letter of word *n* and the subsequent space. Prior to the eyes crossing this boundary, the preview of word *n* + 1 was either (1) identical to that word (e.g., *garden*), (2) a wordlike nonword (e.g., *puvtur*), or (3) a nonwordlike nonword (e.g., *xbtchp*). This resulted in a 2 (frequency) × 3 (preview) design. Both the frequency and preview conditions were fully counterbalanced for subjects and items.

The wordlike previews had a higher bigram frequency than the nonwordlike previews (see Table 1). The display changes in the filler sentences were delayed by 15 ms to ensure that subjects would occasionally see an easily detectable change.

Once readers had crossed the boundary, the preview was replaced by the target word. Custom-made software ensured that display changes during the experimental trials were executed quickly (8 ms, on average). Identical trials were used to estimate false alarm rates for *d’* calculations. In 98 trials a detectable change occurred during reading (68 experimental trials and 30 filler trials), and in 34 trials there was no change.

After each trial, subjects rated (by buttonpress) how confident they were that a display change had occurred, using a 6-point scale (1 = *very confident there was no change*, 6 = *very confident there was a change*). Approximately 50 % of these ratings were followed by a two-alternative comprehension question (answered by buttonpress). Subjects practiced responding to the rating prompts and comprehension questions during ten practice trials, 50 % of which contained display changes. The mean comprehension accuracy was 88.3 % (*SD* = 4.7 %).

## Results

Like Slattery et al. (2011), we calculated hit rates and false alarm rates for each subject, condition, and detection confidence level in the experimental trials. We removed all trials in which there was a blink or track loss on the target word, or in which the saccade only crossed the boundary temporarily before stabilizing to the left of the boundary (about 2 % of trials). We also excluded experimental trials in which the display change finished later than 5 ms after the beginning of the subsequent fixation (about 11 % of trials).

### Display change detection performance

We defined five levels of confidence on the basis of subjects’ confidence ratings. For each level, hits/false alarms were defined as a rating greater than the confidence level, and a miss/correct rejection was a rating equal to or below the confidence level. Trials with a rating above the confidence level were considered false alarms if they were in the identical condition, and hits otherwise. We then used the hit and false alarm rates to compute the *d’* sensitivity for each subject, each nonidentical preview condition (the identical preview condition was used to estimate the false alarm rates), and each detection confidence level by converting the hit and false alarm rates to *z* values and using the formula *d*′ = *z*(*Hit*) − *z*(*FA*).

**Fig. 2:**
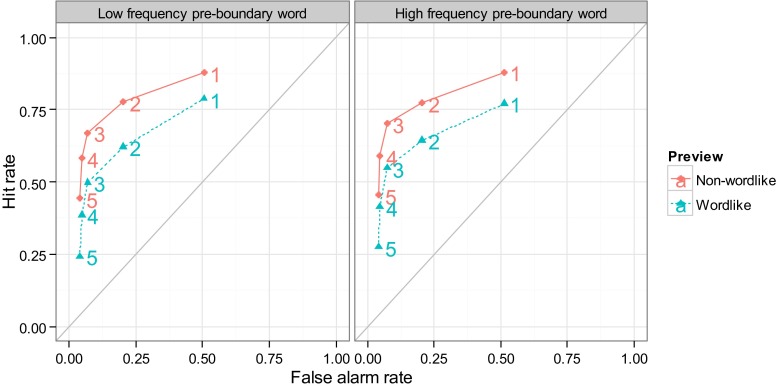
Receiver-operating characteristic curves for the display change detection task in the wordlike and nonwordlike preview conditions. Numbers denote confidence levels. The diagonals are added in gray to aid interpretation

Figure 2 shows receiver-operating characteristic (ROC) curves averaged over subjects for each level of preview, frequency, and confidence. The curves indicate an increase in sensitivity for nonwordlike as compared to wordlike previews, whereas the frequency of the preboundary word *n* does not have an effect or modulate the preview effect. In order to test this statistically, we performed an analysis of variance (ANOVA) on the *d’* values for each subject and condition at Confidence Level 3 (see Table 2). The ANOVA confirmed the significant effect of preview on *d’*, *F*(1, 31) = 50, *η*^2^_G_ = .11, *p* < .01, indicating that nonwordlike previews were associated with greater change detection sensitivity than wordlike previews. However, neither the main effect of preboundary word frequency, *F*(1, 31) = 1.9, *η*^2^_G_ = .0068, *p* > .05, nor the interaction between preview and preboundary word frequency, *F* < 1, approached significance.^1^

**Table 2:** Display change detection sensitivity measures

Preview	Preboundary frequency	Hit rate	False alarm rate	zHit	zFA	*d'*
Nonwordlike	low	.67	.17	0.56	–1.2	1.8
Nonwordlike	high	.68	.18	0.61	–1.2	1.8
Wordlike	low	.51	.17	0.00	–1.2	1.2
Wordlike	high	.53	.18	0.08	–1.2	1.3

### Gaze duration: Preboundary word

Table 3 shows mean gaze durations (GD: the sum of first-pass fixations on a word) on the preboundary word by preview and frequency condition. Using the lme4 package (Bates, Maechler, Bolker, & Walker, 2014) for the R statistical software (R Development Core Team, 2014), we fitted a linear mixed model (LMM) for GD that included fixed effects for word *n* frequency,^2^ word *n* + 1 preview,^3^ and their interaction, as well as random intercepts and slopes by subject and item for each fixed effect.^4^ The LMM results are summarized in Table 4. The preboundary word frequency manipulation had the expected effect: GD was longer for low-frequency (332 ms) than for high-frequency (264 ms) preboundary words (*b* = 0.1, *SE* = 0.011, *t* = 9.49).

**Table 3:** Gaze duration means on the preboundary word

Preboundary frequency	Target preview	GD
Low	Identical	309 (143)
	Wordlike	340 (171)
	Nonwordlike	346 (169)
High	Identical	246 (86)
	Wordlike	267 (118)
	Nonwordlike	281 (151)

**Table 4:** Linear mixed model results for gaze duration on the preboundary word

	GD		
Predictor	Estimate	Std. Error	*t* Value
(Intercept)	5.6004	0.0291	**192.3444**
Target preview (masked vs. identical)	0.0821	0.0220	**3.7286**
Target preview (wordlike vs. nonwordlike)	0.0179	0.0231	0.7753
Preboundary frequency (low vs. high)	0.1026	0.0108	**9.4933**
Target preview (masked vs. identical) * Preboundary frequency (low vs. high)	0.0009	0.0155	0.0577
Target preview (wordlike vs. nonwordlike) * Preboundary frequency (low vs. high)	–0.0251	0.0180	–1.3964

Significant *t* values (|*t*| ≥ 1.96) are printed in **bold**

We found significant parafoveal-on-foveal effects of the preview manipulation on GDs on the preboundary word. We also observed a significant difference between the identical preview condition (278 ms) and the mean of the wordlike (304 ms) and nonwordlike (315 ms) preview conditions (*b* = 0.082, *SE* = 0.022, *t* = 3.73), whereas the difference between the latter two conditions was not significant (|*t*| ≤ 1). Additionally, the interaction between preboundary frequency and preview did not reach significance (|*t|* < 1.4). In summary, we found a robust frequency effect on the preboundary word as well as evidence for orthographic parafoveal-on-foveal effects. However, these parafoveal-on-foveal effects were not modulated by preboundary word frequency. An LMM with Word *n* Frequency as a factor, log token bigram frequency of the preview as a continuous predictor (centered), and their interaction indicated a significant effect of preview bigram frequency on GD on word *n*, with lower bigram frequency resulting in a larger parafoveal-on-foveal effect (|*t*| = 3.35), but again, no evidence for an interaction of preview bigram frequency with word *n* frequency (|*t*| ≤ 0.63).

### Gaze duration: Target word

Table 5 shows the means for GDs on the target word by preview and frequency condition. The LMM results are summarized in Table 6. We observed a main effect of preboundary frequency (*b* = 0.016, *SE* = 0.0073, *t* = 2.26; high frequency, 313 ms; low frequency, 325 ms).

**Table 5:** Gaze duration means on the target word

Preboundary frequency	Target preview	GD
Low	Identical	271 (116)
	Wordlike	342 (138)
	Nonwordlike	360 (141)
High	Identical	241 (87.7)
	Wordlike	345 (135)
	Nonwordlike	348 (128)

**Table 6:** Linear mixed model results for gaze duration on the target word

	GD		
Predictor	Estimate	Std. Error	*t* Value
(Intercept)	5.6784	0.0264	**214.7050**
Target preview (masked vs. identical)	0.3087	0.0284	**10.8869**
Target preview (wordlike vs. nonwordlike)	0.0345	0.0257	1.3463
Preboundary frequency (low vs. high)	0.0164	0.0073	**2.2558**
Target preview (masked vs. identical) * Preboundary frequency (low vs. high)	–0.0451	0.0161	**–2.8051**
Target preview (wordlike vs. nonwordlike) * Preboundary frequency (low vs. high)	0.0139	0.0203	0.6828

Significant *t* values (|*t*| ≥ 1.96) are printed in **bold**

Significant preview benefit effects occurred with shorter GDs in the identical preview condition (256 ms), relative to the wordlike (343 ms) and nonwordlike (354 ms) conditions (*b* = 0.31, *SE* = 0.028, *t* = 10.89). However, the difference between the wordlike and nonwordlike previews was not significant (|*t*| ≤ 1.35).

Additionally, the interaction between preboundary frequency and preview benefit on the target word was significant (*b* = –0.045, *SE* = 0.016, *t* = *–*2.81). For clarity, we report the size of the preview benefit effect (the difference between the means of the wordlike and nonwordlike conditions and the identical condition) rather than the individual condition means. Readers obtained more of a preview benefit when the preboundary word was high in frequency (104 ms) than when it was low in frequency (71 ms). These effects replicate the findings by Henderson and Ferreira (1990). However, preboundary frequency did not modulate the difference between the wordlike and nonwordlike preview conditions (|*t*| ≤ 1).

### Post-hoc analysis: Display change detection effects

The results presented above demonstrate that the properties of the preview had an effect on both GD and display change detection on the preboundary word and the target word. However, the sizes of both the preview benefit effect on the target word and the parafoveal-on-foveal effect on the preboundary word were quite a bit larger than normal. One possible explanation for these large effects is that the display change detection task may have interfered with normal reading behavior. Another possibility is that the larger effects originate in the detection itself. To test these possibilities, we classified each trial on the basis of the display change detection outcome, collapsing over preview type and treating detection ratings of 1, 2, 3, and 4 as “no change detected” responses, and ratings of 5 and 6 (confident and very confident that there was a change) as “change detected” responses. A trial without a display change could then result in either a correct rejection (no change detected) or a false alarm (since false alarms were very rare, they were excluded from further analysis). A trial with a display change could result in either a miss (no change detected) or a hit (change detected). If the large effects obtained in the present experiment are due to its dual-task nature (i.e., performing the secondary detection task changes reading behavior), then these effects should be large whether or not readers detected the changes. However, if the large effects are not the result of looking for a change, but rather are due to finding a change, then the large effect sizes should only be evident in the “change detected” trials. Tables 7 (preboundary word) and 8 (target word) show the mean GD for each of the three detection outcomes in the analysis, as well as for the two preboundary word frequency conditions.

**Table 7:** Gaze duration means on the preboundary word by display change (DC) detection response

Preboundary frequency	Display change detection	GD
Low	Correct rejection (no DC)	309 (143)
Low	Display change missed	310 (139)
Low	Display change detected	377 (192)
High	Correct rejection (no DC)	246 (86)
High	Display change missed	247 (88.9)
High	Display change detected	302 (166)

**Table 8:** Gaze duration means on the target word by display change (DC) detection response

Preboundary frequency	Display change detection	GD
Low	Correct rejection (no DC)	271 (116)
Low	Display change missed	316 (134)
Low	Display change detected	389 (136)
High	Correct rejection (no DC)	241 (87.7)
High	Display change missed	302 (118)
High	Display change detected	391 (128)

We then fitted LMMs on the log preboundary word and target word GDs, with display change detection outcome, preboundary word frequency, and their interaction as predictors. Since display change detection outcome had three levels, we fitted two orthogonal contrasts. Contrast 1 compared trials with correct rejections to trials with misses, whereas Contrast 2 compared the trials in which no display change was detected (i.e., trials with correct rejections and misses) to trials in which display changes were correctly detected (i.e., trials with hits). These LMMs had random intercepts for subjects and items and random slopes for display change outcomes by subject and item. More general models (e.g., including random slopes for frequency by subject) did not converge. The LMM results are summarized in Tables 9 and 10.

**Table 9:** Linear mixed model results for gaze duration on the preboundary word by display change (DC) detection response

	GD		
Predictor	Estimate	Std. Error	*t* Value
(Intercept)	5.591	0.028	**199.560**
DC detection (correct rejection vs. missed)	0.019	0.021	1.597
DC detection (no change detected vs. change detected)	0.107	0.024	**4.245**
Preboundary frequency (low vs. high)	0.104	0.008	**13.972**
DC detection (correct rejection vs. missed) * Preboundary frequency *n* + 1 (low vs. high)	–0.004	0.019	–0.390
DC detection (no change detected vs. change detected) * Preboundary frequency *n* + 1 (low vs. high)	0.011	0.015	1.012

Significant *t* values (|*t*| ≥ 1.96) are printed in **bold**

**Table 10:** Linear mixed model results for gaze duration on the target word by display change (DC) detection response

	GD		
Predictor	Estimate	Std. Error	*t* Value
(Intercept)	5.684	0.024	**240.549**
DC detection (correct rejection vs. missed)	0.206	0.027	**7.560**
DC detection (no change detected vs. change detected)	0.329	0.028	**11.688**
Preboundary frequency (low vs. high)	0.019	0.007	**2.780**
DC detection (correct rejection vs. missed) * Preboundary frequency *n* + 1 (low vs. high)	–0.044	0.017	**–2.571**
DC detection (no change detected vs. change detected) * Preboundary frequency *n* + 1 (low vs. high)	–0.016	0.015	–1.055

Significant *t* values (|*t*| ≥ 1.96) are printed in **bold**

On the preboundary word, we found no significant difference in GDs between correct rejection trials (mean GD = 278 ms) and miss trials (mean GD = 279 ms; *t* = 1.60), indicating that the presence of a display change did not affect fixation times on the preboundary word if it was not detected. However, there was a significant difference between trials on which no display change was detected and trials on which display changes were correctly detected (mean GD = 340 ms; *b* = 0.11, *SE* = 0.027, *t* = 4.24), showing that detecting a display change was associated with a significant cost in terms of fixation time, even on the preboundary word. As expected, we found a significant effect of frequency (*b* = 0.1, *SE* = 0.0074, *t* = 13.97), but none of the interactions of frequency with display change outcome reached significance (*t* = *–*0.39 and 1.01 for Contrasts 1 and 2, respectively).

On the target word, we did find a significant difference in GDs between correct rejection trials (mean GD = 256 ms) and miss trials (mean GD = 310 ms; *b* = 0.21, *SE* = 0.027, *t* = 7.56). This demonstrates that trials on which no display change took place showed a preview benefit relative to trials on which a display change took place and was not detected. Importantly, the size of this preview benefit effect (54 ms) was much smaller than the one reported in the main analysis and is comparable to those in previous studies (e.g., Rayner, 1975). We also observed a significant difference between trials on which no display change was detected and those on which display changes were correctly detected (mean GD = 390 ms; *b* = 0.33, *SE* = 0.028, *t* = 11.69), showing that detecting a display change again was associated with a significant cost in terms of fixation time. This can account for the inflated preview benefit reported in the main analysis. Not surprisingly, given the previous analysis, there was a significant spillover effect of frequency (*b* = 0.019, *SE* = 0.007, *t* = 2.78). The interaction between Contrast 1 and frequency reached significance (*b* = –0.044, *SE* = 0.017, *t* = *–*2.57), suggesting that the foveal load effect (Henderson & Ferreira, 1990) was present even when display changes were not detected. However, there was no interaction between Contrast 2 and frequency (*t* = *–*1.05), indicating that the display change detection effect on fixation times was not modulated by the frequency of the preboundary word.

In summary, our analysis shows that the detection of display changes, rather than the mere task of looking for them, causes inflated fixation times. When we only consider trials on which display changes are present but not detected, we find a standard preview benefit effect and a standard foveal load effect (in relation to the identical preview trials, in which there was no visible display change).

## Discussion

We investigated whether display change detection during reading uses the same cognitive resources as natural reading, by testing (1) whether display change detection uses orthographic regularity and (2) whether display change detection is affected by the processing difficulty of the word preceding the boundary that triggers the display change.

Regarding the first question, we found that subjects were significantly more sensitive to display changes when the change was from a nonwordlike preview than when the change was from a wordlike preview. On the other hand, the preview benefit effect on the target word was not affected by whether the preview was wordlike or nonwordlike.

Regarding the second question, we did not find any influence of preboundary word frequency on display change sensitivity, although eye movement measures indicated foveal load effects (Henderson & Ferreira, 1990), with low-frequency preboundary words reducing the preview benefit that readers obtained for the target word.

Regarding the dual-task nature of our experiment (reading and display change detection) and the larger-than-normal preview and parafoveal-on-foveal effects obtained, post-hoc analyses indicated that these large effects were due to detecting a change rather than looking for a change. These analyses corroborate the findings reported by White et al. (2005), who showed greater preview benefit effects for subjects who were aware of display changes than for subjects who were unaware of display changes, on a more stringent trial-by-trial, within-subjects basis. Therefore, the large preview benefit effects associated with display change detection do not appear to be the result of systematic differences in reading strategies between “detectors” and “nondetectors,” but rather seem to be a consequence of detecting a change.

Our results indicate that display change detection does not use the same cognitive mechanisms involved in parafoveal lexical processing during natural reading. Rather, readers are sensitive to unusual parafoveal letter combinations—that is, parafoveal orthographic information (see also White, 2008). Unusual parafoveal information usually leads to orthographic parafoveal-on-foveal effects, which are frequently observed in gaze-contingent boundary studies (e.g., Angele & Rayner, 2011; Angele, Slattery, Yang, Kliegl, & Rayner, 2008; Angele et al., 2013) and were also found in the present study. Given our results, display change detection and orthographic parafoveal-on-foveal effects may be driven by the same cognitive mechanisms.

Together with the findings by Angele et al., (2013), our results indicate that parafoveal processing occurs in two distinct stages. First, there may be an early “visual check” stage that can influence the duration of the ongoing fixation and is sensitive to orthographic information. Early parafoveal processing may initially operate on a concrete visual representation before transitioning to an abstract letter representation, as was shown by Slattery et al. (2011). Angele et al. (2013) showed that letter identity information is available during this stage and can facilitate processing of the currently fixated word. The purpose of this stage may be to monitor the reading process: Are the eyes fixating close enough to the upcoming word that letters can be recognized, and do these letter combinations look familiar? When the upcoming word looks extremely visually familiar (such as *the*), a skipping saccade may be automatically triggered (Angele & Rayner, 2013). If the upcoming word is not skipped, the preliminary parafoveal information may also help determine the intended within-word saccade target (Hyönä, 1995; Radach, Inhoff, & Heller, 2004; White & Liversedge, 2006a, b). Consistent with our findings on display change detection in the present study, these orthographic effects on initial fixation position do not seem to be influenced by foveal processing difficulty (White & Liversedge, 2006b). In the E-Z Reader model of eye movement control during reading (Reichle, Pollatsek, Fisher, & Rayner, 1998; Reichle, Pollatsek, & Rayner, 2006; Reichle, Warren, & McConnell, 2009), there currently is no direct correspondence to this stage, although there may be some overlap with early visual processing during the “V” stage. Our “visual check” stage is preattentional and may be concurrent with foveal word processing (for an account of how parafoveal information can facilitate foveal processing at this stage, see Angele et al., 2013).

Deeper lexical processing would then occur during a second, attention-dependent stage of parafoveal processing, which corresponds closely to the “L1” and “L2” stages of familiarity check and lexical access in the E-Z Reader model. Our results show evidence for both stages of processing: The initial stage is reflected in the display change detection sensitivity and orthographic parafoveal-on-foveal effects, whereas the second stage is reflected in the preview benefit effect.

In summary, we have shown that the orthographic regularity (“wordlike-ness”) of a preview has effects on display change detection performance and fixations on the preboundary word, independent of ongoing lexical processing. This suggests that parafoveal processing takes place in two stages: an early, orthography-based, preattentional stage, and a late, attention-dependent lexical access stage.

## Footnotes

1 We also used a cumulative link mixed model with a probit link (fitted by the clmm function from the ordinal package for the R statistical software; Christensen, 2014), as recommended by Knoblauch and Maloney (2012). The results agreed with the ANOVA.

2 As a factor. Contrast: low (1) versus high (–1).

3 As a factor. Contrast 1: identical (–1) versus the wordlike (.5) and nonwordlike conditions (.5); Contrast 2: wordlike (–1) versus nonwordlike (1).

4 The random item slopes for frequency and its interaction with preview were removed due to LMM convergence issues.
